# Multiple Dendritic Cell Populations Activate CD4^+^ T Cells after Viral Stimulation

**DOI:** 10.1371/journal.pone.0001691

**Published:** 2008-02-27

**Authors:** Adele M. Mount, Christopher M. Smith, Fiona Kupresanin, Kristina Stoermer, William R. Heath, Gabrielle T. Belz

**Affiliations:** Division of Immunology, The Walter and Eliza Hall Institute of Medical Research, Melbourne, Victoria, Australia; Federal University of São Paulo, Brazil

## Abstract

Dendritic cells (DC) are a heterogeneous cell population that bridge the innate and adaptive immune systems. CD8α DC play a prominent, and sometimes exclusive, role in driving amplification of CD8^+^ T cells during a viral infection. Whether this reliance on a single subset of DC also applies for CD4^+^ T cell activation is unknown. We used a direct *ex vivo* antigen presentation assay to probe the capacity of flow cytometrically purified DC populations to drive amplification of CD4^+^ and CD8^+^ T cells following infection with influenza virus by different routes. This study examined the contributions of non-CD8α DC populations in the amplification of CD8^+^ and CD4^+^ T cells in cutaneous and systemic influenza viral infections. We confirmed that *in vivo,* effective immune responses for CD8^+^ T cells are dominated by presentation of antigen by CD8α DC but can involve non-CD8α DC. In contrast, CD4^+^ T cell responses relied more heavily on the contributions of dermal DC migrating from peripheral lymphoid tissues following cutaneous infection, and CD4 DC in the spleen after systemic infection. CD4^+^ T cell priming by DC subsets that is dependent upon the route of administration raises the possibility that vaccination approaches could be tailored to prime helper T cell immunity.

## Introduction

Murine dendritic cells (DC) can be divided into at least six different subsets [1]. Despite this heterogeneity, in previous studies we have established that the tissue-derived CD8α^+^ (CD8α) DC are the main subset driving CD8^+^ T cell amplification during the early phase of the immune response [2]–[5].

In skin infection, migratory DC (such as Langerhans cells and dermal DC) have long been thought to be key mediators of T cell immunity. We have, however, recently shown that CD8^+^ T cell activation appears to be purely driven by CD8α DC [3]–[5]. This suggests that the long held paradigm that coordinated maturation and migration of peripheral DC to the lymph node is required for T cell activation may be more complicated than first described. In lung infection two distinct DC subtypes–the lymph node-resident CD205^+^CD11b^−^CD8α^+^ (CD8α) DC and the trafficking CD205^+^CD11b^−^CD8α^−^ DC–are responsible for activating naïve virus-specific killer T cells [6]. Nevertheless, even in skin infection with Herpes simplex virus-1 (HSV-1), which does not apparently invoke migratory Langerhans cells directly for immune activation, interplay between these DC transporting antigens and lymph node-resident DC is key to triggering T cell amplification by cross-presentation [6], [7]. These findings suggest a more complex view of viral antigen presentation than first thought. They allude to a more likely scenario whereby co-operation between multiple specialised DC is required to prime naïve T cells.

The role of different DC in major histocompatibility complex (MHC) class II antigen presentation promoting CD4^+^ T cell priming in the setting of an infectious pathogen is poorly understood. This is in part due to the paucity of *in vivo* models that allow simultaneous analysis of the CD4^+ ^and CD8^+^ T cell responses to viral antigens. Despite this, a number of studies have been undertaken to examine naïve CD4^+^ T cell priming using both model and infectious antigens. They have implicated the involvement of CD11b^+^ tissue-derived migratory DC in priming CD4^+^ T cell responses [8], [9] [10]. Subcutaneous (s.c.) injection of the MHC class II-restricted model antigen hen egg lysozyme showed that both CD11c^+^ CD8α DC and CD8α^−^ (non-CD8α) were capable of presenting hen egg lysozyme but CD8α DC were most efficient [10]. By contrast, CD11b^+ ^DC, but not CD8α DC, induced ovalbumin-specific CD4^+^ T cell priming after s.c. administration of soluble ovalbumin [9]. Itano and colleagues [11] employed fluorescently-labelled protein to trace the role of the skin-derived migratory DC in antigen presentation to CD4^+^ T cells. Significantly, Langerhans cells and dermal DC trafficked to the draining lymph nodes on encounter with antigen and both activated naïve T cells resulting in functionally effective CD4^+^ T cells. However, this group reported little involvement of CD8α DC in priming CD4^+^ T cells. Similarly in murine models of Leishmaniasis, a parasitic infection whose control is mediated mainly by CD4^+^ T cells [12], antigen presentation appears to be restricted to CD8α DC [13] and CD11b^+^DC, likely the dermal DC [14]. These studies imply that tissue-derived trafficking DC may play a more important role in activating CD4^+^ T cells, but they do not discount the involvement of CD8α DC.

In this report, we have endeavoured to understand the interplay between different DC subsets in driving T cell activation following subcutaneous and systemic infection with virus. This extends our previous work by highlighting how major and minor DC subsets are involved in both CD8^+^ and CD4^+^ T cell activation. We have evaluated the contribution of tissue-derived trafficking DC, in addition to the role of CD8α DC. We show that, although antigen presentation to CD8^+^ T cells is largely driven by the CD8α DC, antigen presentation to CD4^+^ T cells involved multiple DC populations, highlighting the differential tasks of DC populations in regulating immune responses.

## Results

### CD8α^+^ DC dominate antigen presentation to CD8^+^ T cells during viral infection, while Langerhans cells and dermal DC play minor roles in presentation

**Figure 1:**
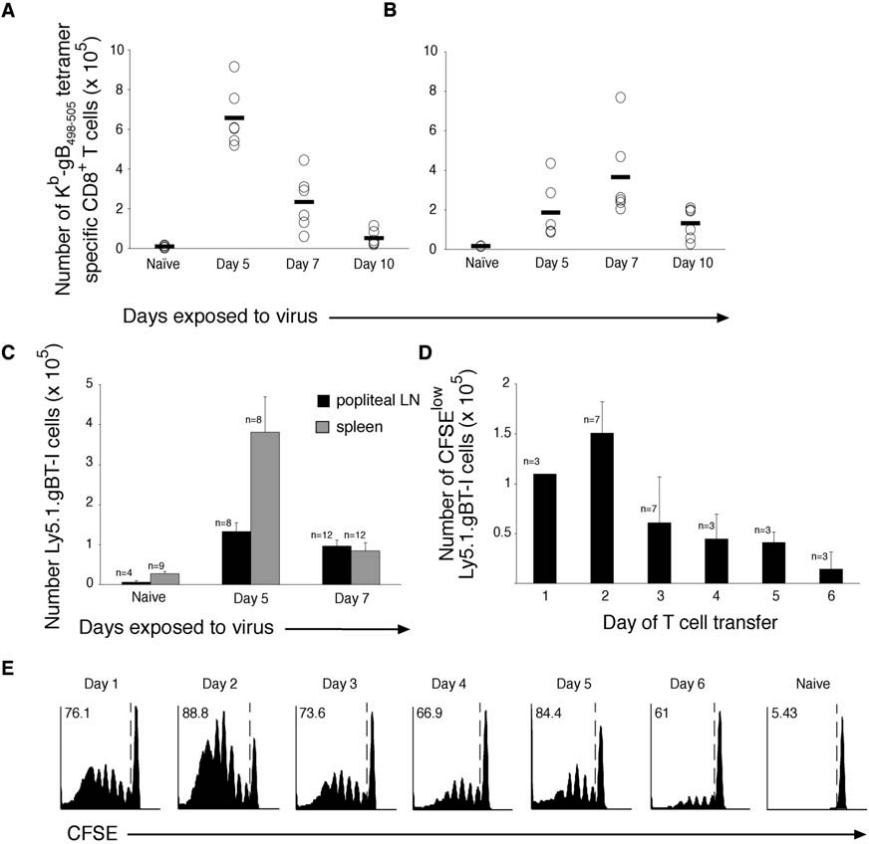
*In vivo* expansion of endogenous and transgenic glycoprotein B-specific CD8^+^ T cells following footpad infection with recombinant influenza WSN-gB. Endogenous gB-specific CD8^+^ T cells were tracked by staining with gB_498–505_ MHC class I tetramers conjugated to the fluorescent dye, phycoerythrin. *A*, The number of gB-specific CD8^+^ T cells were analysed in the popliteal lymph node and *B*, spleen five, seven and ten days after s.c. infection via footpad injection with 400 PFU of recombinant influenza, WSN-gB. Data are pooled from two independent experiments and show the mean of four to six mice at each time point. *C*, 5×10^4^ Ly5.1 gBT-I-specific T cells were adoptively transferred by tail vein injection into naïve C57BL/6 mice one day prior to s.c. infection with 400 PFU of recombinant influenza, WSN-gB. Five and seven days after infection, the number of Ly5.1 gBT-I cells in the spleen was enumerated by flow cytometry by staining for surface expression of CD8α and CD45.1. Data are pooled from two independent experiments and show the mean±SEM of eight to twelve mice at each time point. *D,* Influenza viral antigen stimulates naïve gBT-I CD8^+^ T cells for several days after infection. 2×10^6^ purified CFSE-labelled Ly5.1gBT-I CD8^+^ T cells were adoptively transferred into naïve C57BL/6 mice at various times after s.c. footpad infection with influenza WSN-gB. Proliferation of transferred Ly5.1gBT-I CD8^+^ T cells was assessed in the draining popliteal lymph node 72 h after transfer by evaluating loss of CFSE fluorescence. Data are pooled from two independent experiments and show the mean±SEM. The number of mice examined at each time point are indicated by *n*. *E.* Naïve CFSE-labelled gBT-I CD8^+^ T cells were transferred into mice infected subcutaneously one to six days earlier with WSN-gB. T cell proliferation in the popliteal lymph node was analysed by flow cytometry three days after transfer. The percentage of proliferated cells is shown in the upper left hand corner of each graph. Profiles show representative CFSE proliferation from data shown in *D*.

Subcutaneous inoculation with influenza WSN-gB virus resulted in maximal expansion of endogenous glycoprotein B (gB)-specific CD8^+^ T cells at five days after infection in the popliteal lymph nodes (Figure 1A) and seven days post-infection in the spleen (Figure 1B). Such a priming approach gave an effective cytotoxic T cell response when monitored by *in vivo* cytotoxicity thirty days after infection indicating that memory T cells with cytotoxic function are generated in this system (data not shown). The recombinant influenza WSN-gB contains the HSV-1 glycoprotein B CD8^+^ T cell epitope engineered into the neurominidase stalk. This allowed us to take advantage of the gB-specific T cell receptor transgenic as a tool to probe the antigen presenting capacity of individual subsets of DC. To determine whether TCR transgenic cells are amplified *in vivo* in a manner analogous to the endogenous gB-specific CD8^+^ T cells, we followed the expansion of gBT-I CD8^+^ T cells after s.c. infection with WSN-gB. The expansion of TCR transgenic cells in spleen appeared to peak two days earlier than for endogenous virus-specific cells (Figure 1C). Similarly, the maximal expansion of transgenic cells in the popliteal lymph nodes 72 h after transfer occurred slightly earlier than that of endogenous gB-specific T cells detectable by MHC tetrameric staining (Figure 1D), possibly reflecting the initial high input precursor frequency of transgenic gB-specific cells in the latter system.

Previously we have examined which DC populations drive amplification of naïve CD8^+^ T cells by direct *ex vivo* analysis acutely after s.c. infection with influenza WSN-gB or HSV [4], [5]. Although this is not a natural route of infection, it does reflect the pathway of pathogen accidentally gaining access to the dermis through injury to the protective barrier of the skin. We used this route to determine whether our previous observation that skin DC did not present HSV-1 viral antigens reflected a feature of the HSV virus, or alternately, reflected a broader characteristic of subcutaneous viral infection [3]. In this situation, we targeted the peak of antigen presentation for analysis but it is not clear whether the small window of antigen presentation examined reflected the full spectrum of DC subpopulations involved in delivering immunogenic signals driving amplification of T cells. To determine how the functional expression of the surrogate influenza epitope, SSIEFARL, derived from the glycoprotein B of HSV, impacted on the level and duration of activation and expansion of CD8^+^ T cells, mice were infected s.c. with WSN-gB and at various times after infection CFSE-labelled transgenic T cells specific for the gB epitope were adoptively transferred. Three days after transfer, popliteal lymph nodes and spleen cells were harvested to examine proliferation of transgenic T cells by flow cytometry (Figure 1C,D,E). The results indicated that maximal antigen presentation occurred in the popliteal lymph nodes two days after infection and decreased over the next four days.

**Figure 2:**
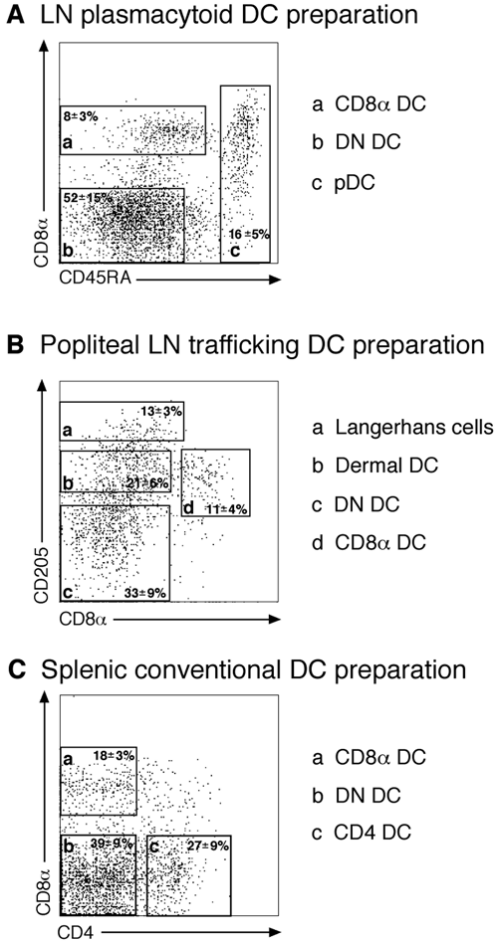
DC were isolated from the popliteal lymph node or spleen of mice and flow cytometrically purified into DC subsets according to one of three sorting protocols. *A*. In the first approach, CD11c^+^ cells were sorted from the popliteal lymph node or spleen into CD8α^+^ (CD8α) DC, CD8α^+/−^CD45RA^+^ plasmacytoid DC (pDC) and CD8α^−^CD45RA^−^ double negative (DN) DC. *B.* In the second approach, CD11c^+^ cells from popliteal lymph node were further dissected into CD8^−^CD205^high^ Langerhans cells, CD8α^−^CD205^int^ dermal DC, CD8α^−^CD205^−^ DN DC and CD8α^+^CD205^int^ DC. *C*. In the third approach, CD11c^+^ cells were sorted from light density cells isolated from spleen into CD8α^+^CD4^−^, CD8α^−^CD4^+^ and CD8α^−^CD4^−^ DC.

**Figure 3:**
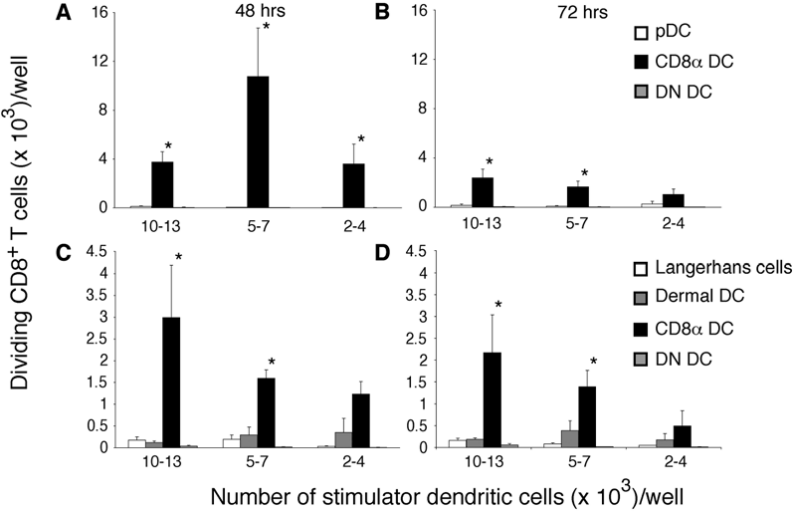
CD8α DC dominate antigen presentation to naïve gBT-I CD8^+^ T cells following s.c. infection. C57BL/6 mice were infected with WSN-gB by s.c. injection into the footpad. 48 and 72 h after infection, the popliteal lymph node were isolated and non-DC lineages were removed by antibody depletion. *A,B.* CD11c^+^ DC subsets were purified by flow cytometric sorting into DN DC, CD8α DC and pDC before culturing titrating numbers of each subset with 5×10^4^ CFSE-labelled gBT-I CD8^+^ T cells. Proliferation was analysed at 60 h of culture. Data show the mean and SEM of three independent experiments. *C,D.* Lymph node-resident CD8α DC and not skin-derived trafficking DC, prime naïve CD8^+^ T cells following s.c. infection with WSN-gB. C57BL/6 mice were infected with 400 PFU by s.c. injection into the footpad. At 48 h (*C*) and 72 h (*D*) following infection, DC were enriched from popliteal lymph node and flow cytometrically sorted into Langerhans cells (CD205^+^CD8α^+/−^), dermal DC (CD205^int^CD8α^−^), CD8α DC (CD205^+^CD8α^+^) or DN DC (CD205^−^CD8α^−^). Titrating numbers of purified DC subsets were cocultured with 5×10^4^ CFSE-labelled gBT-I CD8^+^ T cells. The x-axis shows the range of DC used as stimulators over different experiments. Proliferation was analysed at 60 hr of culture. Data are pooled from three independent experiments and show the mean and SEM. Significant differences (*p*≤0.05) in T cell proliferation induced by different DC populations are indicated by an asterisk.

In earlier studies which suggested that CD8α DC were the sole DC subset responsible for presentation of influenza antigen, analysis of antigen presentation was restricted to segregation of DC populations into CD8α DC, CD8α^−^ DC (termed double-negative (DN) DC) and plasmacytoid DC (pDC) (Figure 2A, 3A,B) [4]. As DN DC are composed of multiple DC subsets including CD205^−^CD11b^+^, Langerhans cells and dermal DC (Figure 2B), we questioned whether weak presentation by individual subsets might have been overlooked. To examine more closely whether the migratory subsets of DC could be involved in the presentation of antigen to CD8^+^ T cells, particularly later in infection when Langerhans cells may have had an opportunity to migrate to the draining lymph nodes [15], we extended our analysis to include these DC subsets at 48 and 72 h after infection (Figure 3C,D). DC were sorted in two ways: either as previously into (i) CD8α DC, DN DC and pDC or (ii) Langerhans cells (CD11c^+^CD205^high^), dermal DC (CD11c^+^ CD8α^low/–^ CD205^intermediate^), CD8α DC (CD11c^+^ CD8α^+^ CD205^intermediate^) and DN DC (CD11c^+^CD8α^–^ CD205^–^) (Figure 2A,B). Building on previous findings using the three-way subdivision of DC (Figure 3A,B), CD8α DC were found to be the dominant APC. When pDC were excluded and DC were additionally separated into conventional subsets, however, weak, but reproducible responses were seen for Langerhans and dermal DC (Figure 3C,D). This suggested that while CD8α DC predominate in viral antigen presentation, other DC subsets may contribute to the development of the immune response.

### Tissue-derived DC dominate activation of naïve CD4^+^ T cell following subcutaneous influenza infection

**Figure 4:**
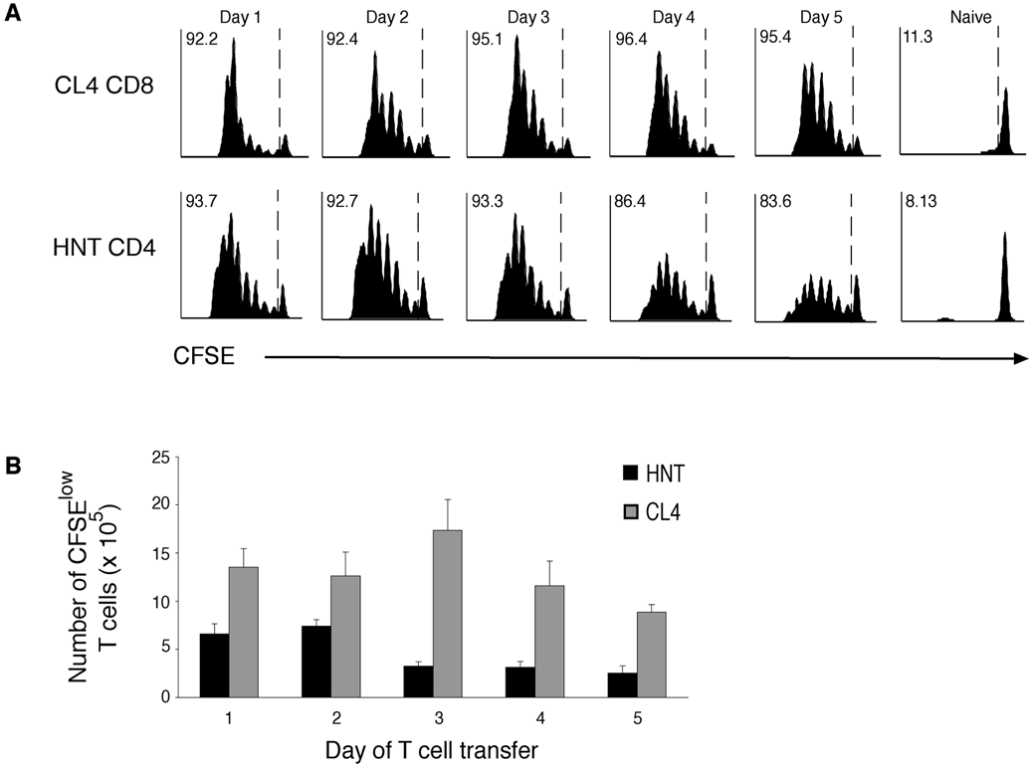
Highly efficient antigen presentation to CD4^+^ and CD8^+^ T cells is limited to the early phase of infection following s.c. viral infection. 2×10^6^ CFSE-labelled purified CD4^+^ and CD8^+^ T cells specific for the HA epitopes of influenza were adoptively transferred into naïve BALB/c mice infected on days 1 to 5 respectively with PR8 influenza virus. Proliferation of T cells in the popliteal lymph node was indicated by dilution of the CFSE stain 3 days after transfer. *A.* Data show representative flow cytometric profiles of T cell proliferation from popliteal lymph node from one of two similar experiments and *B,* Histogram shows the mean number of dividing T cells in popliteal lymph node±SEM 3 days after transfer. Data are pooled from two experiments showing 5 mice for HNT CD4^+^ expansion and 6 mice for CL4 CD8^+^ expansion.

Given that Langerhans cells and dermal DC do not appear to play a major role in activating CD8^+^ T cells during s.c. infection, we hypothesised that these subsets may be more important in driving activation of CD4^+^ T cells. To extend our observations into a system that would enable us to simultaneously investigate the contribution of antigen presentation by different DC subsets to both CD4^+^ and CD8^+^ T cells, we utilized influenza infection in BALB/c mice. In this model, we were able to take advantage of the MHC class II and MHC class I-restricted T cell receptor transgenic mice that recognize antigenic determinants encoded in influenza haemaglutinin (HA). This allowed us to simultaneously track the response of both CD4^+^ and CD8^+^ T cells to the same viral antigen. Firstly, we determined the *in vivo* kinetics of antigen presentation driving expansion of HA-specific CD8^+^ (CL4) and CD4^+^ (HNT) T cell responses (Figure 4A,B). The pattern of T cell activation was similar to that observed for s.c. infection with WSN-gB infection in C57BL/6 mice with strong proliferation observed for CD4^+^ and CD8^+^ T cells peaking around days two and three.

**Figure 5:**
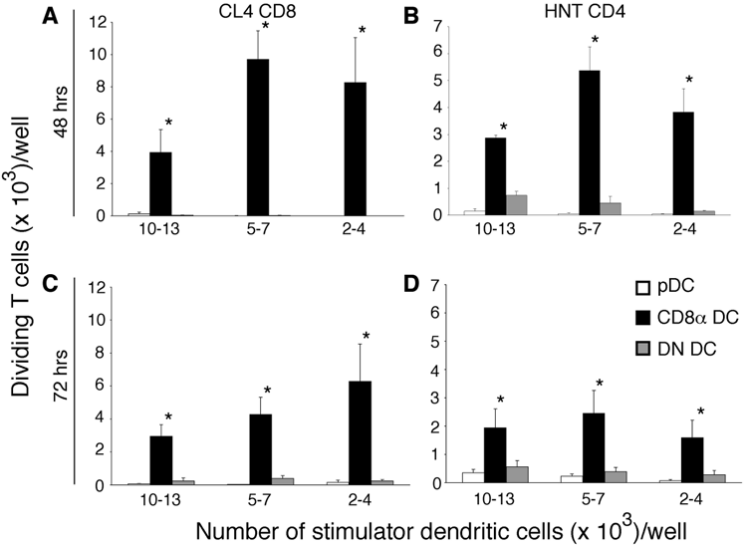
Multiple subsets of DC present influenza HA antigen to T cells in BALB/c mice infected with PR8 virus by s.c. injection into the footpad. The popliteal lymph nodes were isolated and non-DC lineages were removed by antibody depletion. CD11c^+^ DC subsets were purified by flow cytometric sorting into DN DC, CD8α DC and pDC before culturing titrating numbers of each subset with 5×10^4^ CFSE-labelled influenza-specific CD8^+^ T cells (*A,C*) or CD4^+^ T cells (*B,D*) 48 (*A,B*) and 72 (*C,D*) h after infection. The x-axis shows the range of DC used as stimulators over different experiments. Proliferation was analysed at 60 h of culture. Data are pooled from (*A–C*) three and four (*D*) independent experiments and show the mean and SEM. Significant differences (*p*≤0.05) in proliferation induced by different DC populations are indicated by an asterisk.

**Figure 6:**
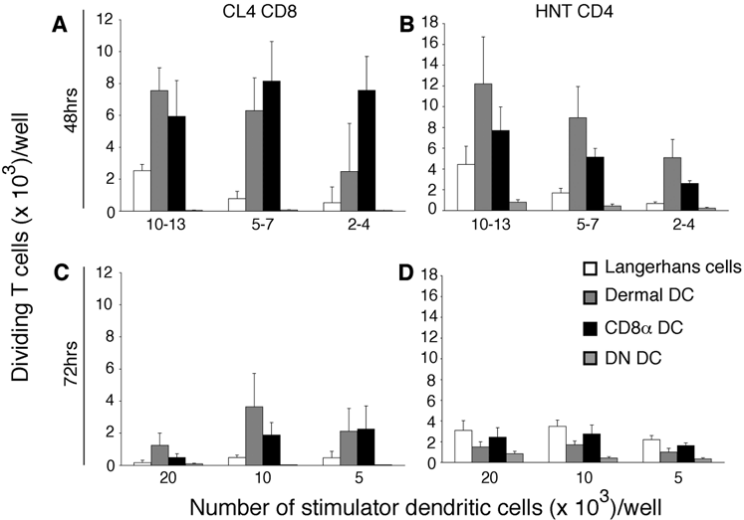
Lymph node-resident and tissue-derived trafficking DC prime naïve influenza HA-specific CD8^+^ and CD4^+^ T cells following s.c. infection with influenza PR8. Naïve BALB/c mice infected with influenza virus (PR8) by s.c. injection. 48 (*A,B*) or 72 h (*C,D*) after infection, DC were enriched from the popliteal lymph nodes and flow cytometrically purified into Langerhans cells (CD11c^+^ CD205^+^), dermal DC (CD11c^+^ CD205^int^), CD8α (CD11c^+^ CD8^+^) DC or DN (CD11c^+^CD8^−^CD205^−^) before culturing titrating numbers of purified DC with 5×10^4^ CFSE-labelled CD8^+^ (CL4) or CD4^+^ (HNT) T cells specific for the HA epitopes of influenza. The x-axis shows the range of DC used as stimulators over different experiments (*A,B*) or the number of DC (*C,D*) used in each well. Proliferation was analysed at 60 hr of culture. Data represent the mean±SEM of four (*A,B*) and three (*C,D*) independent experiments.

To examine antigen presentation by different DC subsets in response to PR8 virus, BALB/c mice were inoculated by s.c. infection and popliteal lymph nodes were harvested two or three days later for DC purification. In the first set of experiments, DC were separated on the basis of CD11c, CD8α and CD45RA expression into CD8α DC, pDC (CD11c^+^CD45RA^+^CD8α^−^ DC) and DN DC and examined for their capacity to present MHC class I and II antigens at both 48 (Figure 5A,B) and 72 h (Figure 5C,D) after infection. This initial separation strategy was used primarily to examine presentation by pDC and CD8α DC (Figure 2A). This revealed that CD8α DC were the major population responsible for both MHC class I and II presentation, and that pDC were unable to prime virus-specific T cells. While negligible responses were seen for DN DC (containing migratory DC) their capacity to stimulate naïve virus-specific T cells could not be excluded based on earlier findings in the C57BL/6 system (Figure 3). To better examine the capacity of migratory DC to present viral antigen in this model, DC were depleted of pDC and separated into the four conventional subsets examined earlier (Figure 6). As in Figure 2*B*, conventional DC were separated into CD8α DC, Langerhans cells, dermal DC and those DC not expressing either CD8 or CD205 (now referred to as DN DC) (Figure 6A–D). Strikingly, this form of separation revealed a major role for dermal DC in both MHC class I and MHC class II priming in BALB/c mice. This is in contrast to the relative absence of antigen presentation detected by DN DC when the unfractionated population was analysed (Figure 5A,B). This approach highlights the importance of fine dissection of DC subsets, particularly when broad phenotypic separation is used in the first instance.

### Broad MHC class II-restricted antigen presentation by multiple DC subsets to CD4^+^ T cells is maintained following intravenous infection

**Figure 7:**
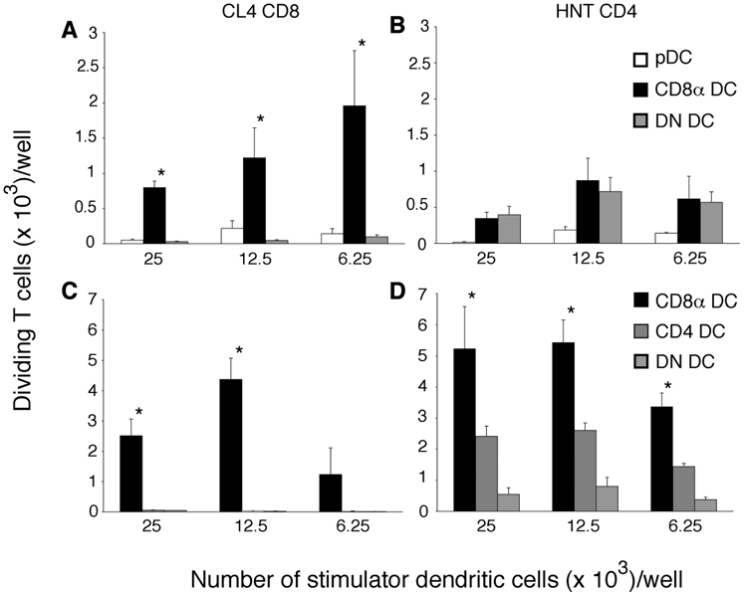
Dendritic cell subsets mediating MHC class I and class II-restricted antigen presentation differ following intravenous infection. *A,B,* 24 h after infection with PR8 virus, non-DC were depleted from spleens and the remaining cells were purified by flow cytometry into CD8α DC (CD8α^+^CD45RA^−^), CD4 DC (CD4^+^CD45RA^−^), and pDC (CD8α^−^CD4^−^CD45RA^−^); or *C,D* alternately, CD8α DC (CD8α^+^CD4^−^), CD4 DC (CD8α^−^CD4^+^) or DN DC (CD8α^−^CD4^−^) subsets. Titrating numbers of each subset was cocultured with 5×10^4^ CFSE-labelled HA-specific CD8^+^ or CD4^+^ T cells and the amount of proliferation was measured at 60 h of culture by the loss of CFSE fluorescence. Data show the mean±SEM of three (A,C) to four (B,D) independent experiments. Significant differences (p≤0.05) in proliferation induced by different DC populations are indicated by an asterisk.

In earlier studies, we used the three-way pDC sort to identify the CD8α DC as the primary subset involved in amplifying CD8^+^ T cell responses to influenza WSN-gB after i.v. infection [4]. To determine whether a similar skewing of DC capacity to prime CD8^+^ T cell responses extended to other influenza viruses and to explore whether this was also the case for CD4^+^ T cells we analysed the ability of pDC, CD8 DC and DN DC to prime influenza-specific MHC class I (CL4) and class II (HNT) in mice infected by intravenous (i.v.) inoculation two days previously with influenza PR8. This approach showed that although CD8 DC were the major DC presenting antigens to CD8^+^ T cells (Figure 7A), multiple subsets of DC, namely the CD8 DC and DN DC presented MHC class II antigens equally efficiently to CD4^+^ T cells (Figure 7B). This strong antigen presenting ability of DN DC, which consists of more than one DC subset, prompted us to further subdivide this population into its respective subsets. The DC from mice infected with PR8 i.v. were depleted of pDC and separated into CD8α^+^CD4^−^, CD8α^−^CD4^+^ and CD8α^−^CD4^−^ DC (Figure 2C) and cocultured with HA-specific CD4^+^ or CD8^+^ T cells. This confirmed that CD8α DC were the sole DC subset responsible for presenting viral antigens to naïve CD8^+^ T cells, but importantly revealed that CD4 DC, and to a lesser extent, DN DC were also involved in driving activation of CD4^+^ T cells (Figure 7C,D).

These data show that after i.v. infection, viral antigen presentation to CD8^+^ T cells is effected primarily by the CD8α DC while MHC class II antigen presentation needed to ensure activation of CD4^+^ T cells, is performed by multiple DC subsets.

## Discussion

The signals and interactions that drive effective DC priming of T cells resulting in protective immunity remain mostly undefined. Although much research has focused on the dual capacity of CD8α DC to promote the induction of tolerance and immunity to pathogens through amplification of CD8^+^ T cells, it remains unclear to what extent MHC class II presentation by CD8α DC is important for the amplification of CD4^+^ T cells in viral infections [3], [5], [16], [17].

DC possess highly effective mechanisms to capture, transport and process antigens. This allows them to present either endogenous viral antigens synthesised within the cell or exogenous antigens captured from other infected cells. *In vitro*, all DC subsets tested can present viral antigens, such as influenza or HSV antigens [4]. In contrast, *in vivo*, CD8α DC resident within the lymphoid tissues appear to be almost solely responsible for driving the activation of CD8^+^ T cells [2]–[5]. Such a universal restriction of both MHC class I and II antigen presentation to a single DC subset in pathogen infection is likely to significantly limit the capacity to activate lymphocyte populations. Many viruses have mechanisms that interfere with antigen presentation pathways and infection of DC offers an opportunity to exploit such mechanisms to promote survival of the virus by substantially crippling the immune response itself. Such a targeting strategy is utilized by lymphocytic choriomeningitis virus (LCMV, clone 13) to selectively infect CD205^+^ DC through the α-dystroglycan receptor, effectively disabling any capacity to generate cytotoxic T cells [18], [19]. To circumvent such a situation, it is likely that other DC subsets are conscripted to activate CD4^+^ T cells, and possibly B cells, during an immune response to pathogens. This has provoked our detailed assessment of the role of different subsets of DC in activating CD4^+^ and CD8^+^ T cells in viral infections.

Cutaneous immune function requires skin-derived antigen presenting cells, such as Langerhans cells and dermal DC, to convey antigenic signals from the periphery and activate CD8^+^ T cells. Several recent studies of viral infections have challenged this paradigm suggesting that lymph node-resident DC, namely the CD8α DC, and not Langerhans cells, drive amplification of CD8^+^ T cell responses, while non-CD8α DC have been purported to play a dominant role in transporting antigen to the lymph node rather than direct priming of T cells [2], [3], [5]. This was based on the lack of antigen presentation by Langerhans cells and dermal DC in cutaneous HSV infection [3]. However, intranasal infection with influenza virus or HSV-1 results in both CD11b^−^ and CD8α DC effectively presenting viral antigens to CD8^+^ T cells [6]. Similarly, lentiviruses, appear to readily infect skin-derived DC and retain their capacity to present viral antigens after trafficking suggesting that both the virus and the antigen influence antigen presentation by different DC subsets [2], [6], [20].

Recent studies addressing the identity of the DC population(s) responsible for initiating CD4^+^ T cell immune responses against pathogens have generally supported a predominant role for non-CD8α DC with only scant evidence for CD8α DC involvement [8], [13], [14], [21]. For example, immunity to Leishmania infection is largely driven by antigen presentation of the Leishmania analogue of the receptors of activated C kinase, known as LACK, antigen by a tissue-derived CD11b^+^ DC that was present in the draining lymph node as early as 2 days after infection [13]. The involvement of CD11b^+^ cells in responses against Leishmania was confirmed by Lemos *et al*. [14] in a study that however also provided evidence of involvement of the CD8α DC. More recently, Leon *et al.* [21], showed that *in situ* differentiation of CD11b^+^ monocyte-derived DC driven by inflammatory mediators could control the induction of protective responses to *L. major*. Similar to Leishmania infection, analysis of herpes simplex virus type 2 (HSV-2) infection of the vagina revealed dermal DC as the major antigen-presenting population activating CD4^+^ T cells in the draining lymph node [8]. These studies give the impression that MHC class II antigen presentation to CD4^+^ T cells might be largely restricted to non-CD8α DC. In infection with *Toxaplasma gondii,* CD8α DC play a clearly established role in MHC class II presentation [22]. In this case, the capture of the immunodominant antigen of the parasite, profilin, is augmented by TLR 11 expression on CD8α DC. Antigen presentation by dermal DC directly to CD8^+^ T cells has not been obvious in several infectious settings. However, in our examination of s.c. WSN-gB infection in C57BL/6 mice, careful separation of DC into Langerhans cells and dermal DC revealed only a small population of non-CD8α DC that could be important in the immune response.

Discrepancies between reports have been noted in analysing the specific role of different DC populations in viral, bacterial or parasitic infections [3], [5], [8], [13], [14], [23]. These differences could arise in part from differences between the experimental models employed, or could alternately, be attributable to differences in the efficiency with which different DC subsets present on MHC class I and class II molecules [24]–[28]. To exclude such variables from our system and to expand our analysis of the contribution of different DC subsets to viral infection, we took advantage of the availability of HA-specific CD4^+^ and CD8^+^ T cells to probe antigen presentation in influenza virus in BALB/c mice *in vivo*. This approach allowed us to examine the ability of different DC subsets to activate both CD4^+^ and CD8^+^ T cells responding to the same viral protein. In our studies we clearly showed that both CD8α and non-CD8α DC play a central role in presenting MHC class I and II antigens to T cells in generating immunity.

Previous studies of DC antigen presentation in the viral setting have suggested a generalized dichotomy in which CD8α and non-CD8α DC are specialized for MHC class I and II antigen presentation, respectively. Our study provides the first direct assessment of MHC class I and class II antigen presentation to the same protein in a viral system that we know of to date. In earlier work, we showed that priming of virus-specific cytotoxic T cells depended on presentation of cognate antigens on MHC class I and II molecules by the same DC implying that CD8α DC, at least, are also involved in ensuring effective CD4^+^ T cell responses [5]. It is now clear that a broader array of DC types within the DC network can effectively present to CD4^+^ T cells. Within the lymphoid tissues, non-CD8α DC have been shown to be most efficient at presenting exogenous antigens on MHC II molecules, while CD8α DC are highly efficient at presenting exogenous antigens on MHC I molecules [27], [29]. Recently, Dudziak et al. [29] elegantly demonstrated that the preferential association of CD8α and non-CD8α DC subsets with priming of CD8^+^ and CD4^+^ T cells to the model antigen ovalbumin reflected the differential expression of genes important for MHC class I and II antigen processing and presentation pathways between the DC populations. This suggests that DC subsets possess intrinsic properties that allow them to handle exogenous antigens selectively for presentation to T cells. However, all DC subsets appear to be able to present endogenously synthesized viral antigens such as from influenza or HSV infection [4], [30], [31]. During influenza infection *in vivo*, however, two subsets of DC predominantly present viral antigens raising the possibility that non-CD8α DC, such as the migratory ones, are more easily accessed by infecting virus. Importantly, these DC can present antigens on MHC class II, and MHC class I molecules, but in addition, they may transport viral antigens to lymphoid-tissue resident CD8α DC, that are specialized to take up exogenous antigens and preferentially present these antigens to CD8^+^ T cells. This capacity to differentially present antigens by different DC may be important in promoting effective naïve and memory T cell responses [32].

Collectively, our results suggest that although CD8α DC are a key DC population involved in priming CD8^+^ T cell responses, targeting of both CD8α and non-CD8α DC subsets may be required to ensure effective long lasting protective immunity.

## Materials and Methods

### Mice

C57BL/6 (B6) (H-2^b^), BALB/c (H-2^d^), gBT-I (H-2K^b^-restricted glycoprotein B, gB_498–505_) [33], CL4 (H-2K^d^-restricted anti-influenza hemagglutinin, HA_512–520_) [34] and HNT (IA^d^-restricted anti-HA_126–138_) [35] mice were bred and maintained in specific pathogen-free conditions at the animal facilities of the Walter and Eliza Hall Institute of Medical Research (Melbourne, Australia). Experiments with all mice began when mice were six to ten weeks of age and were performed in accordance with guidelines of the Melbourne Directorate Animal Ethics Committee.

### Virus Infections

Mice were anaesthetized with methoxyfluorane and then infected with influenza virus diluted in 20 µl of PBS for footpad infection. For footpad infection either 10^2.6^ PFU recombinant influenza WSN-gB (H1N1) which contains the gB_498–505_ K^b^-restricted epitope of HSV inserted into the neurominidase stalk [36] was used, or 10^3.9^ A/Puerto Rico/8/34 (Mt. Sinai, H1N1) (PR8) influenza A virus was used. For i.v. infection 10^4.9^ PR8 virus was diluted in 200 µl of PBS and injected into the tail vein.

### Preparation of CFSE-labelled transgenic T cells 

Peripheral lymph nodes (inguinal, axillary, brachial, sacral, superficial cervical and mesenteric) were obtained from CD4^+^ (HNT) mice or CD8^+ ^(CL4 or gBT-I.1) TCR transgenic mice and purified using a cocktail of optimally titred antibodies to deplete cells expressing Mac-1 (M1/70), Mac-3 (F4/80), Ter-119, GR1 (RB6-8C5), MHC class II (M5/114) and either CD8 (53.6.7) or CD4 (GK1.5) followed by sheep anti-mouse and anti-rat dynabeads (Dynal). Enriched cells contained 87–96% specific TCR transgenic T cells. These were labelled with 5,6-carboxyfluorescein diacetate succinimidyl ester (CFSE; Molecular Probes) by incubating 10^7^ purified cells per ml with 5 µM CFSE for 10 min at 37°C. Cells were then washed three times in hepes modified Eagles medium containing 2.5% FCS (HEM2.5).

### Analysis of virus-specific CD8^+^ T cell numbers by PE-streptavidin conjugated K^b^-gB tetramer

To quantify the number of viral antigen-specific T cells, single cell suspensions from spleen and lymph nodes were firstly enriched for CD8^+^ T cells using magnetic bead depletion with the antibodies M1/70 (anti-Mac-1), F4/80, Ter 119, RB6-8C5 (anti-Gr-1), M5/114 (anti-class II MHC) and GK 1.5 (anti-CD4) followed by goat anti-rat IgG-coupled magnetic beads (Dynal, Oslo and Qiagen).

Enriched CD8^+^ cells were washed and stained with PE-streptavidin conjugated K^b^-gB tetramer containing the immunodominant HSV gB_498–505 _peptide for 45 minutes at room temperature. Cells were then stained with APC conjugated anti-CD8 mAb (53-6.7) for 30 minutes on ice in a balanced salt solution containing 2% FBS. PI staining was used to exclude dead cells (2 µg/mL; Sigma) and data was acquired on at least 10^4^ viable cells using a FACSCalibre flow cytometer (BD Biosciences). Data was analysed using CellQuest Pro (BD Biosciences).

### Isolation of DC from lymph node and spleen

DCs were isolated essentially as described [15], [37]. Briefly, the draining lymph nodes or spleen fragments were digested for 20 min at room temperature with collagenase/DNase (1 mg/ml collagenase type II, Worthington Biochemicals, Lakewood, NJ; and 1 µg/ml grade II bovine pancreatic DNase I, Boehringer-Mannheim, Mannheim, Germany) and then treated for 5 min with EDTA to disrupt T cell-DC complexes. Cells not of the DC lineage were depleted by incubating in predetermined optimal concentrations of purified Abs: anti-CD3 (KT3), anti-Thy1 (T24/31.7), anti-CD19 (ID3), anti-GR-1 (RB6-8C5), and anti-erythrocyte (TER-119) and then removing the Ab-binding cells with anti-rat Ig-coupled magnetic beads (Dynabeads; Dynal, Oslo, Norway). Note that in our hands pDC are not depleted using anti-GR-1 mAb (19, 20). The DCs in the enriched populations were gated as CD11c^+^ cells before sorting into specific subsets by fluorescence activated cell sorting (MoFlo instrument; Cytomation, Fort Collins, CO). For lymph node, DC were sorted into CD8α^+^, CD45RA^+^ and DN DC or alternatively, CD8α^low/−^CD205^high ^(Langerhans cells), CD8α^high^CD205^−^ (conventional CD8α^+^ DC), CD8α^−^CD205^int^ (dermal DC) and CD CD8α^−^CD205^−^ (DN DC). In our hands staining pDC with CD45RA faithfully recapitulates staining with more recently developed antibodies such as pPDCA-1, therefore CD45RA was used to maintain consistency with previous studies. Post-sorting analysis of sorted DC populations showed that purity ranged from 90–99% and the average purity of populations used for stimulation were 98%.

### Analysis of *in vitro* proliferation of naïve T cells by DC

5×10^4^ enriched CFSE-labelled CD4^+^ or CD8^+^ TCR transgenic cells were added to graded numbers of flow cytometrically sorted DC in 200 µl RPMI 1640 containing 10% FCS, 50 µM 2-mercaptoethanol, 2 mM L-glutamine, 100 U/ml penicillin and 100 µg/ml streptomycin in 96-well V-bottom plates (Costar®, Corning Incorporated, NY). Cultures were analysed for proliferation after 60 hr. Cells were harvested and stained with anti-CD4-APC or anti-CD8α-APC (GK1.5, 53-6.7; BD Pharmingen) and resuspended in 150 µl flow cytometry buffer containing 2.5×10^4^ Callibrite beads (6 µm; Becton Dickinson). Samples were analysed by flow cytometry by on PI^−^ exclusion until 5×10^3^–1×10^4^ beads were collected. Differences in proliferation induced by different subsets of DC were analysed using the Student's t-test. Statistically significant comparisons, *p*≤0.05, are denoted with an asterisk.

### Analysis of antigen presentation in vivo 

Mice inoculated with influenza virus or left untreated and 2×10^6^ CFSE-labelled transgenic cells were adoptively transferred into recipients by i.v. injection. Then, 60–72 h later, mice were killed and gBT-I, HNT or CL4 proliferation in popliteal lymph node or spleen was determined by flow cytometry.
